# 

**Published:** 1916-03-04

**Authors:** 


### Re-examination of Rejected Recruits.

AN unusually large proportion of the time of the House of Commons has been devoted to matters of medical interest arising not only out of questions addressed to Ministers, but in the course of the debates. On no less than three days of the week the subject of the re-examination of men who had been rejected for the Army as medically unfit has been the subject of prolonged discussion, and the matter has also been referred to in the House of Lords. The most notable occasion was that on which Sir John Simon, in the course of the finance debate, criticised the action of recruiting authorities. He stated that men had had their rejection certificates torn up before their eyes, and had then been re-examined and passed into the ranks; and he instanced some remarkable cases in which men alleged to be in an advanced stage of unfitness had been re-examined and passed into the Army. It is not necessary in these columns to make further allusion to the debates on this matter, and it will suffice to add that the Under-Secretary for War has undertaken that any man who has been the victim of "trickery" will be put back into the place he was in before. Mr. Tennant admitted, in reply to a question by Mr. Booth, that many consumptives had enlisted, but as soon as it was discovered that a man was suffering from phthisis he was invalided from the Service.

### GIFTS AND BEQUESTS.

Mr. Walter Williams, of Eastbourne, left £250 to the London Hospital.

The Fleming Memorial Hospital, Newcastle, has received a gift of £1,000 from Mr. Cecil Cochrane.

Mrs. Ellen Constance Soames, of Porchester Terrace, left £100 to the Children's Hospital, Worth Court, Hampstead.

The Fishmongers' Company has made a grant of £105 to the Rebuilding Fund of the Chelsea Hospital for Women.

Miss Helen Fletcher, of Nottingham, has bequeathed £200 to the Nottingham General Hospital and £100 each to nine other charities.

Miss Emma Price, of Tunbridge Wells, who died at the age of eighty-eight, bequeathed £200 to the Royal Albert Hospital, Devonport.

Miss Ellen Pearce, of Southsea, left £500 each to the Portsmouth, Portsea, and Gosport Hospital and the Portsmouth Victoria Association for Nursing the Sick Poor.

The Manchester Royal Infirmary has received £250 from Mr. Charles Hopkinson, a member of the board. This is the second instalment of a donation from Mr. Hopkinson to endow a bed.

The Edinburgh Royal Infirmary has received an anonymous donation of £1,800 in Five per Cent. Exchequer Bonds, to endow a bed to be called "The Philpstoun Oil Works, Linlithgow, Bed."

Mr. George Wheeler, of Canonbury Park South, has bequeathed £200 each to the London Orphan Asylum, Watford, and the British Orphan Asylum, Slough, and £100 each to the London Fever Hospital and the Great Northern Hospital, Holloway.

The Rev. Dr. Stuart Alexander Donaldson, D.D., Master of Magdalene College, Cambridge, since 1904, and formerly Vice-Chancellor of the University, left £100 to Addenbrooke's Hospital.

Sir Ernest Cassel, whose gifts to hospitals have won universal gratitude, has given £3,200 in 4½ per cent. War Stock to the London Hospital, where a ward has been named the "Maud Ashley Ward" in memory of his daughter.

The promoters of the London Jewish Hospital have received gifts amounting to £1,500, being £1,000 presented by three sons of the late Mrs. Marian Levy, in memory of their mother, conditional on five other sums of £100 each being received. It has been decided to name the medical consulting room after Mrs. Marian Levy.

Over £100 has been realised for Ancoats Hospital from a benefit performance on its behalf given by the proprietors of the Royal Osborne Theatre, Machester. This is the sixteenth year that these benefits have been given. The Workpeople's Fund Committee of the hospital undertake all the arrangements, and have displayed great energy in carrying them out successfully. This year's receipts brought the total from this source to considerably over £1,500.

The Right Hon. Arnold Morley, a former Postmaster-General, who died on January 17, left £2,500 each to the General Hospital, Nottingham, the Convalescent Homes Association, Nottingham, and the Children's Hospital, Nottingham; £1,000 to the Women's Hospital, Nottingham; £500 each to the Anglo-American Hospital, Cairo, the Seamen's Hospital Society, and the South London Association for Assisting the Blind. These are additional to numerous other bequests to charity.

### PASSING EVENTS AND TOPICS.

### St. George's Hospital.

A Special Court of the Governors of St. George's Hospital will be held at the hospital on Wednesday, March 8, at 2.45 p.m., to elect a treasurer.

### Women House Surgeons.

The Royal Portsmouth Hospital is endeavouring to obtain the services of two women house surgeons. The salary offered for each appointment is £150 a year, with board, etc.

### The London Hospital.

Sir Bertrand Dawson, Mr. Lister, Mr. Lett, and Mr. Souttar are away on service with the Forces. Twenty-six London Hospital nurses have been supplied to the Navy, and 120 to the Army.

### Royal Medical Benevolent Fund Guild.

At the annual meeting of this organisation held at the Mansion House on February 21, the financial statement, presented by Dr. Mary Scharlieb, showed an income of £3,726, inclusive of a legacy of £5C0, and receipts to the amount of £1,000 as a result of a sale held at Crewe House.

### An Interesting Endowment.

A sum of over £700 has been handed to the Ilford Emergency Hospital for the endowment of a bed as a permanent memorial to the passengers that were killed in the railway accident that occurred at Ilford in January 1915, and as a thank-offering from some of those surviving. The fund was organised by relatives of some of the deceased passengers, who formed themselves into an Accident Fund Committee, under the chairmanship of Mr. Parker. A tablet recording the circumstances of the gift has been put up in the hospital.

### A Lost-Limb Bureau.

An example of Germany's matter-of-fact thoroughness is to be found in the establishment at Charlottenburg of what may appropriately be termed a lost-limb bureau (*Gliederersatz-prüfstelle*). Its purpose is to supply artificial arms and legs and to determine the class of work for which individuals may be suited when supplied with these. Soldiers from the various hospitals will be sent to this bureau, and, according to the Berlin correspondent of the official organ of the American Medical Association, a school is to be founded in connection with the bureau in which maimed soldiers will be taught suitable trades.

RATES OF SUBSCRIPTION: Twelve months, 6/6; Six months, 4/-; Three months, 2/-, post free to any address in the United Kingdom. Abroad, Twelve months, 8/8; Six months, 5/-; Three months, 2/6, post free.—Address The Subscription Dept., "THE HOSPITAL," The Hospital Building, 28/29 Southampton Street, Strand, London, W.C.

